# Accuracy of bone mineral density quantification using dual-layer spectral detector CT: a phantom study

**DOI:** 10.1007/s00330-017-4801-4

**Published:** 2017-04-03

**Authors:** Robbert W. van Hamersvelt, Arnold M. R. Schilham, Klaus Engelke, Annemarie M. den Harder, Bart de Keizer, Harald J. Verhaar, Tim Leiner, Pim A. de Jong, Martin J. Willemink

**Affiliations:** 1 Department of Radiology, University Medical Centre Utrecht, P.O. Box 85500, 3508 GA Utrecht, The Netherlands; 2 Institute of Medical Physics, University of Erlangen-Nürnberg, Erlangen, Germany; 3 Department of Nuclear Medicine, University Medical Centre Utrecht, Utrecht, The Netherlands; 4 Department of Geriatric Medicine, University Medical Centre Utrecht, Utrecht, The Netherlands

**Keywords:** Dual-energy CT, Dual-layer spectral detector CT, Dual energy X-Ray absorptiometry, Bone mineral density, Material decomposition

## Abstract

**Objectives:**

To investigate the accuracy of bone mineral density (BMD) quantification using dual-layer spectral detector CT (SDCT) at various scan protocols.

**Methods:**

Two validated anthropomorphic phantoms containing inserts of 50–200 mg/cm^3^ calcium hydroxyapatite (HA) were scanned using a 64-slice SDCT scanner at various acquisition protocols (120 and 140 kVp, and 50, 100 and 200 mAs). Regions of interest (ROIs) were placed in each insert and mean attenuation profiles at monochromatic energy levels (90–200 keV) were constructed. These profiles were fitted to attenuation profiles of pure HA and water to calculate HA concentrations. For comparison, one phantom was scanned using dual energy X-ray absorptiometry (DXA).

**Results:**

At both 120 and 140 kVp, excellent correlations (R = 0.97, P < 0.001) were found between true and measured HA concentrations. Mean error for all measurements at 120 kVp was -5.6 ± 5.7 mg/cm^3^ (-3.6 ± 3.2%) and at 140 kVp -2.4 ± 3.7 mg/cm^3^ (-0.8 ± 2.8%). Mean measurement errors were smaller than 6% for all acquisition protocols. Strong linear correlations (R^2^ ≥ 0.970, P < 0.001) with DXA were found.

**Conclusions:**

SDCT allows for accurate BMD quantification and potentially opens up the possibility for osteoporosis evaluation and opportunistic screening in patients undergoing SDCT for other clinical indications. However, patient studies are needed to extend and translate our findings.

***Key points*:**

• *Dual*-*layer spectral detector CT allows for accurate bone mineral density quantification*.

• *BMD measurements on SDCT are strongly linearly correlated to DXA*.

• *SDCT*, *acquired for several indications*, *may allow for evaluation of osteoporosis*.

• *This potentially opens up the possibility for opportunistic osteoporosis screening*.

## Introduction

Osteoporosis is a disease associated with low bone mineral density (BMD), increasing the risk for fractures, and thereby contributes substantially to morbidity and mortality and carries social and economic burdens [1]. Bone mineral density is found to be an independent predictor for future fracture risk and all-cause mortality [2, 3]. Early detection and treatment of osteoporosis by using BMD measurements can contribute to the prevention of osteoporosis-related fractures [3].

BMD derived from dual energy X-ray absorptiometry (DXA) is the preferred method for the assessment of osteoporosis according to the World Health Organisation [4]. DXA is widely implemented due to its low costs and low radiation exposure. However, particularly in elderly subjects, the use of the projection DXA technique, which measures an areal BMD (aBMD) in g/cm^2^, is sensitive for errors. There is the size dependency of an areal BMD measurement; artefacts which cause inaccuracies (overlying soft tissue, aortic calcifications, vertebral fractures and spinal degenerative changes); and integral bone measurements as DXA measures the whole vertebra including the neural arch, thereby including the cortical bone [5], whereas the inner trabecular bone is found to be more metabolically active and thus more influenced by changes in bone mineral density [6]. Quantitative computed tomography (QCT) is unaffected by these problems, and in addition also allows for a differentiation of cortical and trabecular bone [6]. However, QCT is subject to higher radiation exposure and typically requires an in-scan calibration phantom, eliminating the option for routine BMD measurements in CT acquired for any indication. Recent studies proposed the use of CT numbers on regular clinically obtained conventional CT scans for the use of BMD assessment with varying sensitivity and specificity of 62–93% and 79–97%, respectively, when compared to DXA [7–10]. In addition, asynchronous BMD calibration using a separate phantom calibration scan, and internal BMD calibration using the paraspinal muscle and subcutaneous fat as reference materials, have been proposed [10]. However, bone density measurements on conventional single-energy CT can be substantially affected by scanner instability, X-ray tube voltages, intravenous contrast medium injection, presence of fat within bone marrow, beam hardening artefacts, patient scatter and metal artefacts [5, 10–14].

With the use of dual energy computed tomography (DECT) artefacts can be reduced and beam hardening can be eliminated [15]. More importantly, with DECT materials can be distinguished by using material decomposition (MD) algorithms [15]. During the eighties DECT for the assessment of BMD was explored by several research groups [12, 13, 16–19]. This allowed for BMD measurements with minimal influence of bone marrow fat [12, 13]. However, mainly due to increased radiation dose of the DECT and an improved precision of single-energy CT protocols, DECT was abandoned. However, DECT was revived and became commercially available a decade ago [20–23]. Since then BMD has been evaluated with dual source DECT [20–22] and rapid kVp switching DECT [23]. For both DECT approaches, a specific DECT protocol has to be selected before scanning the patient, eliminating the option for retrospective BMD measurements when DECT was not selected.

Recently a novel DECT technique has become commercially available that enables retrospective dual energy analyses on all scan protocols when images are acquired at 120 or 140 kVp. The novel dual-layer spectral detector CT (SDCT) uses a single tube with a dual-layer detector separating low and high energy X-ray photons to reconstruct dual energy images. In addition, a conventional image for clinical use is always reconstructed from the data by combining the output of both layers, eliminating the need to select a DECT protocol beforehand. This could potentially open up the possibilities for retrospective BMD evaluation and opportunistic screening for osteoporosis on all SDCT scans acquired for regular clinical indications and thereby potentially eliminating the need for an additional DXA scan. However, to the best of our knowledge BMD has not yet been assessed on SDCT and the accuracy is thus unknown. Therefore, the aim of this study was to evaluate the accuracy of SDCT for quantification of BMD using various CT protocols.

## Materials and methods

### Phantoms

**Fig. 1:**
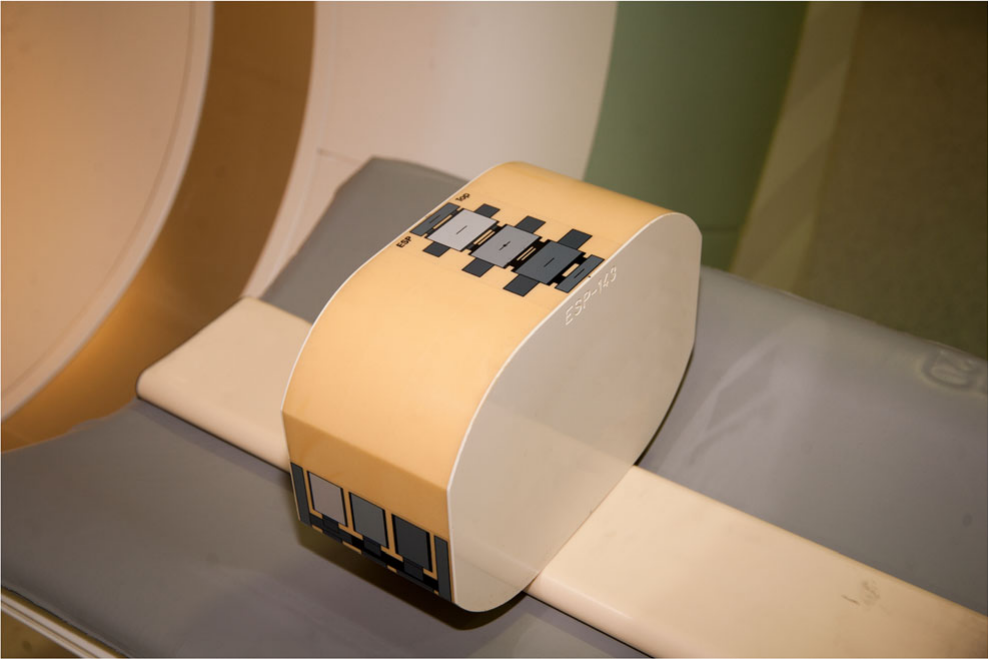
Phantom setup. An anthropomorphic European spine phantom on top of a bone density calibration phantom

An anthropomorphic European spine phantom (ESP; QRM GmbH, Moehrendorf, Germany) on top of a bone density calibration phantom (BDC; QRM GmbH, Moehrendorf, Germany) was imaged on a SDCT system (Fig. 1). Subsequently the ESP was scanned on a DXA system. The ESP resembles the lumbar region of a small adult person and is used as standard for quality control in CT and DXA [24]. The phantom consists of water-equivalent resin containing three vertebral inserts with different BMD quantities. BMD is defined as the amount of calcium hydroxyapatite (HA) or Ca_5_[OH|(PO_4_)_3_] per volume unit of bone. The ESP contains three different inserts with trabecular bone compartments containing nominal design values of 50, 100 and 200 mg/cm^3^ HA to mimic a (patho)physiological range of BMD. Measured on DXA these vertebral inserts represent an areal BMD (aBMD) of 0.5, 1.0 and 1.5 g/cm^2^ HA, respectively. The BDC phantom contains three cylindrical inserts with a diameter of 18 mm containing a nominal design value of 0, 100 and 200 mg/cm^3^ HA. The specific ESP phantom (ESP-143) and BDC phantom (BDC-03-29) used in this study consisted of 51.2, 102.3 and 201.2 mg/cm^3^ HA and 0, 104.4 and 206.2 mg/cm^3^ HA, respectively. For clarity, the nominal design values are used in the remainder of the text, whereas the latter phantom specific concentrations were used as true HA concentration for determining accuracy. The manufactured accuracy of these certified HA values is 1% [24]. In standard single-energy QCT the BDC phantom allows for calibration of the measured Hounsfield units (HU) to BMD, which are then applied to the subject (replaced in this case by the ESP). However, in our DECT setup the BMD calibration is no longer required; instead the BDC was used as an additional check to test the accuracy of BMD quantification on SDCT.

### SDCT image acquisition and reconstruction

The phantoms were scanned with a novel 64 detector row SDCT system (iQon Spectral CT, Philips Healthcare, Best, The Netherlands). The SDCT uses a single beam and a single detector with two layers in which the top layer detects low-energy X-ray photons and the bottom layer high-energy X-ray photons. This information is used to reconstruct spectral-based images (SBI), allowing for dual energy options. In addition, conventional images are automatically reconstructed by combining the information detected on both layers. Images were acquired in spiral mode at 120 and at 140 kVp. Tube current time product was set to 50, 100 and 200 mAs to simulate various abdominal, chest and spine SDCT imaging protocols, resulting in a volumetric CT dose index (CTDI_vol_) of, respectively, 4.5, 9.0 and 18.1 mGy at 120 kVp and 6.5, 13.0 and 26.0 mGy at 140 kVp. Dose length product (DLP) was calculated by multiplying the CTDIvol with the scan length of 12.3 cm. An estimation of the effective dose was calculated by multiplying the DLP by a conversion coefficient of 0.0153 mSv/[mGy•cm] for 120 kVp acquisitions and 0.0155 mSv/[mGy•cm] for 140 kVp acquisitions (Table 1) [25]. CT data were obtained using 64x0.625 mm collimation, pitch 0.925 and gantry rotation time 0.4 s.

**Table 1:** Scan protocols

kVp	mAs	CTDIvol (mGy)	DLP (mGy · cm)	ED (mSv)
140	200	26.0	319.8	5.0
120	200	18.1	222.6	3.4
140	100	13.0	159.9	2.5
120	100	9.0	110.7	1.7
140	50	6.5	80.0	1.2
120	50	4.5	55.4	0.8

*CTDIvol* volumetric CT dose index, *cm* centimetre, *DLP* dose length product, *ED* effective radiation dose, estimated as DLP · k (k 0.0153 for 120 kVp and k 0.0155 for 140 kVp), *kVp* kilovoltage peak, *mAs* milliampere second, *mGy* milligray, *mSv* millisievert

The raw projection data from both detector layers were reconstructed into SBI with standard filter B using spectral level 0. Spectral reconstruction is a model-based iterative reconstruction algorithm developed for SDCT with levels ranging from 0 to 6, whereby a higher level implies more noise reduction. In addition to spectral level 0, spectral levels 1–6 were reconstructed to analyse the influence of iterative reconstruction on BMD quantification. Subsequently, images at monochromatic X-ray photon energies were formed based on the raw data in the projection space. Slice thickness and increment were both 3 mm. Reconstructed images were analysed on a dedicated workstation (IntelliSpace Portal v6.5.0.02080, Philips Healthcare).

### SDCT image analysis and BMD quantification

**Fig. 2:**
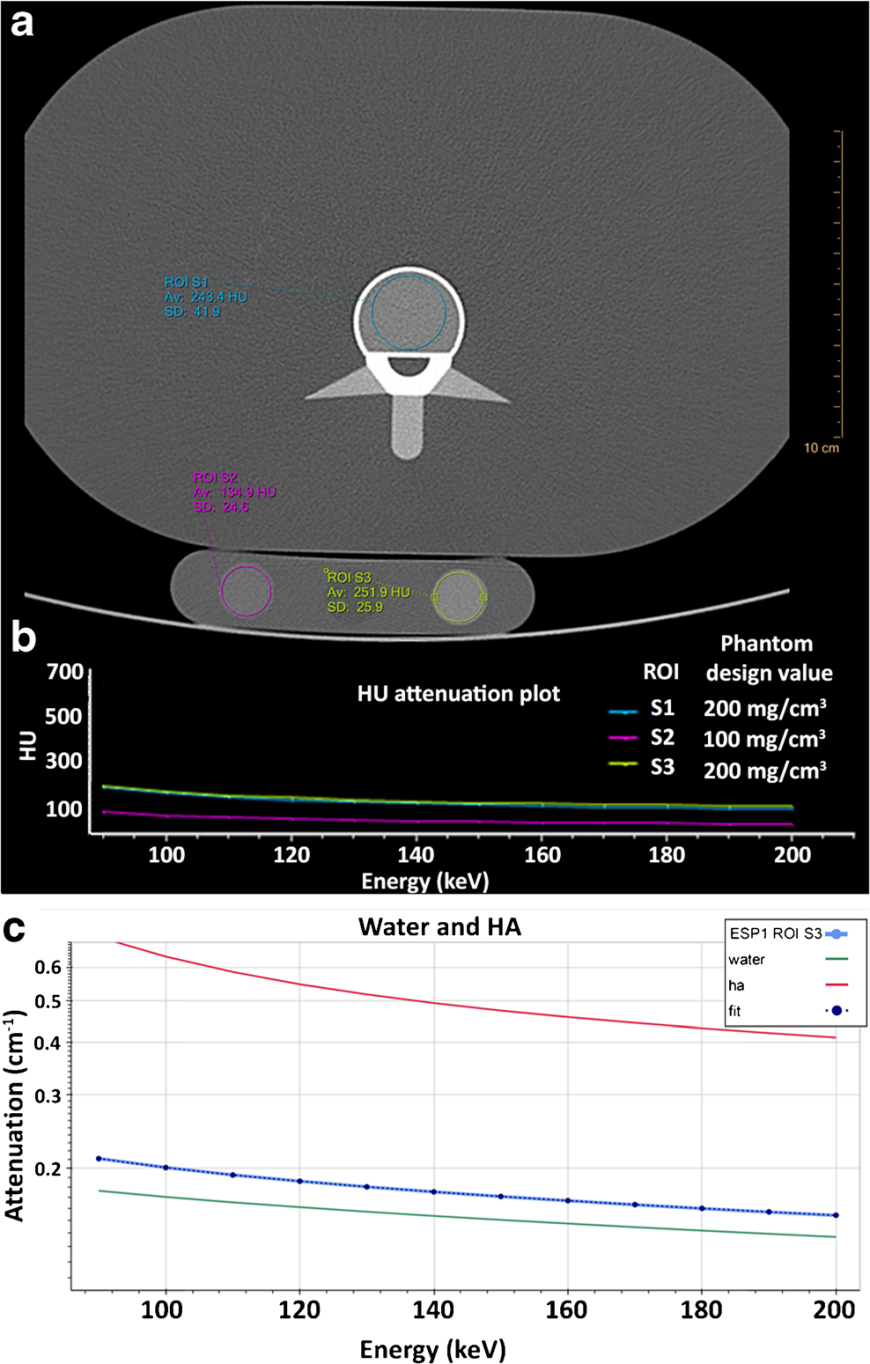
Image analyses and bone mineral density quantification. (**A**) Axial image with a ROI drawn in the ESP (blue) and BDC (pink and green). (**B**) Spectral plot for the corresponding ROIs: mean HU versus monochromatic energy level (keV). (**C**) Attenuation profiles were constructed between 90 and 200 keV in steps of 10 keV. Using in-house developed software, HA concentrations were calculated by fitting the constructed profiles to known attenuation profiles of pure HA and pure water. For this image this concerns ROI S3, an insert with a phantom design value of 200 mg/cm^3^ HA concentration. *Av* average, *BDC* bone density calibration phantom, *ESP* European spine phantom, *HA* calcium hydroxyapatite, *HU* Hounsfield units, *keV* kilo electron voltage, *ROI* region of interest, *SD* standard deviation

On the ESP images, a circular region of interest (ROI) with a fixed area of 450 mm^2^ was analysed in the centre of the trabecular bone of the vertebral insert (Fig. 2A). On the BDC images, a ROI with a fixed area of 150 mm^2^ was analysed in the centre of the insert (Fig. 2A). The water insert of the BDC was not used. For each scan, ROIs of three different slices per (vertebral) insert in both the ESP and BDC scans were analysed. For each ROI, mean HU were plotted against monochromatic energy levels between 90 and 200 keV (Fig. 2B). This range was chosen to suffer the least from potential metal artefacts [26, 27]. In steps of 10 keV, profiles of mean attenuation were constructed. So far the manufacturer software does not derive BMD from the monochromatic images. Therefore BMD values were calculated using in-house developed software by fitting the constructed attenuation profiles to known attenuation profiles of HA and water (Fig. 2C).The known attenuation profiles were obtained from the National Institute of Standards and Technology (NIST) database [28]. For this method no calibration phantom was needed.

For the purpose of the analysis, results from the three slices were averaged per corresponding insert. In addition, the results of the 100 and 200 mg/cm^3^ HA inserts of the ESP and BDC were also combined. Thus three, six and six individual measurements per scan contributed to the average results of the 50, 100, and 200 mg/cm^3^ inserts, respectively.

### DXA image acquisition and BMD quantification

For comparison, the ESP was also scanned on a DXA system (Hologic Discovery A, Hologic Inc., Bedford, MA, USA) using standard settings routinely used in clinical practice. Images of the vertebral inserts were acquired in posterior-anterior position. A rectangular ROI was manually drawn over each vertebral insert to segment the bone region and quantify the aBMD in g/cm^2^. For the comparison between SDCT and DXA only the ESP measurements were used.

### Statistical analysis

The primary study outcome measure was accuracy expressed as measurement errors of the investigated method on SDCT. Measurement errors were defined as the difference between the measured HA and the true HA concentration known from the phantom, expressed in mg/cm^3^:

$$ \mathrm{Measurement}\kern0.5em \mathrm{error}\kern0.5em \left(\mathrm{mg}/{\mathrm{cm}}^3\right)=\mathrm{Measured}\kern0.5em \mathrm{H}\mathrm{A}\kern0.5em \mathrm{concentration}-\mathrm{H}\mathrm{A}\kern0.5em \mathrm{concentration} $$

Relative accuracy errors were obtained in %:

$$ \mathrm{Relative}\kern0.5em \mathrm{measurement}\kern0.5em \mathrm{error}\kern0.5em \left(\%\right)=\frac{\mathrm{measurement}\kern0.5em \mathrm{error}\kern0.5em \left(\mathrm{mg}/{\mathrm{cm}}^3\right)}{\mathrm{true}\kern0.5em \mathrm{HA}\kern0.5em \mathrm{concentration}\kern0.5em \left(\mathrm{mg}/{\mathrm{cm}}^3\right)}\times 100 $$

All measurements performed at 120 and 140 kVp were analysed separately. Spearman’s rho correlation coefficient between measured and true HA concentration was determined for all measurements. The Shapiro-Wilk test was used to evaluate normality of the data. Statistical differences of all measurement errors between 120 and 140 kVp were analysed using the paired t-test, comparing the mean of the two samples of related data. Statistical differences of measurement errors between different scan protocols and different iterative reconstruction levels were analysed using the repeated measures analysis of variance (ANOVA) and post hoc pairwise comparison with Bonferroni correction. Mauchly’s test was used to test the assumption of sphericity. The Greenhouse-Geisser correction was used when this assumption was violated. The correlation between measured HA concentrations on SDCT and aBMD measured on DXA was evaluated using linear regression analyses. Values are listed as mean with standard deviation (SD), unless stated otherwise. A P-value <0.05 was used to indicate statistical significance. IBM SPSS version 21.0 (IBM corp., Armonk, NY, USA) was used for statistical analyses.

## Results

**Fig. 3:**
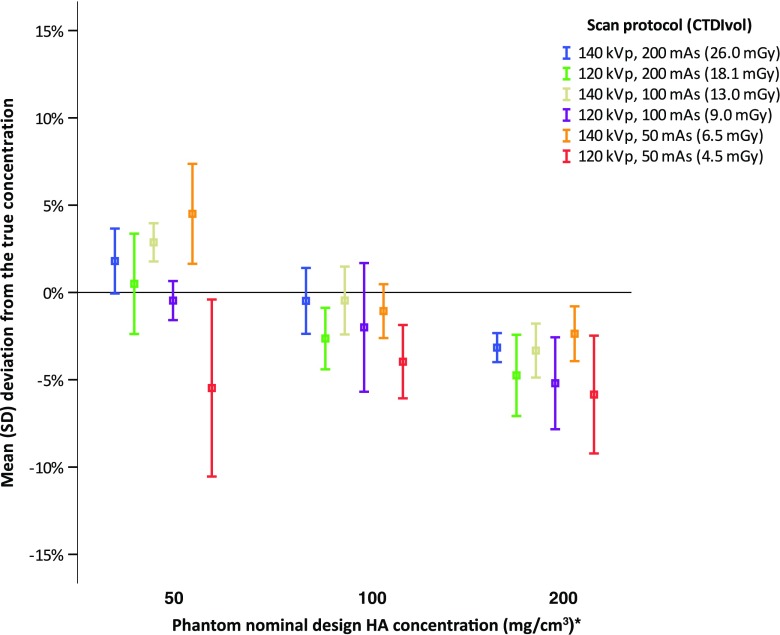
Accuracy of dual-layer detector spectral CT for bone mineral density quantification. Mean relative errors (%) per protocol per nominal design HA concentration are shown. Symbols indicate mean measurement error (%) and error bars standard deviation. * = Phantom specific concentrations (ESP-143; 51.2, 102.3 and 201.2 mg/cm^3^ HA and BDC-03-29; 104.4 and 206.2 mg/cm^3^ HA) were used to determine deviation. *CTDIvol* volumetric CT dose index, *ESP* European spine phantom, *HA* calcium hydroxyapatite, *kVp* kilovoltage peak, *mAs* milliampere second, *mGy* milligray, *SD* standard deviation

At both 120 and 140 kVp excellent correlations (R = 0.97 and 0.97, respectively, P < 0.001) were found between true and measured HA concentrations. The mean error for all measurements (N = 90) was -4.0 ± 5.0 mg/cm^3^ (-2.2 ± 3.3%). The mean error for all measurements at 120 kVp (N = 45) was -5.6 ± 5.7 mg/cm^3^ (-3.6 ± 3.2%) and was significantly (P < 0.001) more deviated from the true concentration than the mean measurement error at 140 kVp (N = 45) of -2.4 ± 3.7 mg/cm^3^ (-0.8 ± 2.8%). At the 50 mg/cm^3^ HA insert, overall mean measurement errors at 120 kVp were more accurate than overall mean measurement errors at 140 kVp, while at 100 and 200 mg/cm^3^ HA inserts overall mean measurement errors at 140 kVp were more accurate (Table 2). When analysing all scan protocols separately, HA quantifications were overall accurate with relative mean measurement errors smaller than 6% for all scan protocols (Fig. 3). Highest relative measurement errors were obtained for the protocol with the lowest dose, 120 kVp 50 mAs. At the 50, 100 and 200 mg/cm^3^ HA inserts relative measurement errors for this lowest dose protocol were -5.5 ± 5.1%, -4.0 ± 2.1% and -5.8 ± 3.4%. Repeated measures ANOVA with Greenhouse-Geisser correction showed a significant difference between measurement errors of different scan protocols (F = 9.62, P < 0.001)). Post hoc tests revealed that measurement errors at the lowest dose protocol (120 kVp 50 mAs) were significantly higher than at 140 kVp 200 mAs (P = 0.013), 140 kVp 100 mAs (P = 0.016) and 140 kVp 50 mAs (P = 0.025), respectively. No significant differences were found between the other protocols. For all scan protocols, no significant differences were found between BMD measurements at different IR levels; repeated measures ANOVA with Greenhouse-Geisser correction were P > 0.05.

**Table 2:** Mean measurement errors. Subdivided by nominal design concentrations, mAs and kVp

True BMD concentration (mg/cm^3^)	mAs	120 kVp		140 kVp	
		Measurement error		Measurement error	
		mg/cm^3^	%	mg/cm^3^	%
50*	All	-0.9 ± 2.1	-1.8 ± 4.0	1.6 ± 1.1	3.1 ± 2.1
	200	0.3 ± 1.5	0.5 ± 2.9	0.9 ± 1.0	1.8 ± 1.9
	100	-0.2 ± 0.6	-0.5 ± 1.1	1.5 ± 0.6	2.9 ± 1.1
	50	-2.8 ± 2.6	-5.5 ± 5.1	2.3 ± 1.5	4.5 ± 2.9
100*	All	-3.0 ± 2.7	-2.9 ± 2.6	-0.7 ± 1.8	-0.7 ± 1.7
	200	-2.7 ± 1.8	-2.6 ± 1.8	-0.5 ± 2.0	-0.5 ± 1.9
	100	-2.1 ± 3.8	-2.0 ± 3.7	-0.5 ± 2.0	-0.5 ± 1.9
	50	-4.1 ± 2.2	-4.0 ± 2.1	-1.1 ± 1.6	-1.1 ± 1.5
200*	All	-10.6 ± 5.3	-5.3 ± 2.7	-6.0 ± 2.7	-2.9 ± 1.3
	200	-9.6 ± 4.6	-4.7 ± 2.3	-6.4 ± 1.6	-3.2 ± 0.8
	100	-10.5 ± 5.2	-5.2 ± 2.6	-6.7 ± 3.1	-3.3 ± 1.5
	50	-11.8 ± 6.7	-5.8 ± 3.4	-4.8 ± 3.1	-2.4 ± 1.6

Data are given as mean ± standard deviation. For 50 mg/cm^3^ nine measurements and for 100 and 200 mg/cm^3^ 18 measurement were performed at both 120 and 140 kVp (three and six,respectively, per mAs value)

*BMD* bone mineral density, *kVp* kilovoltage peak, *mAs* milliampere second

* Phantom specific concentrations (ESP-143; 51.2, 102.3 and 201.2 mg/cm^3^ HA and BDC-03-29; 104.4 and 206.2 mg/cm^3^ HA) were used to determine measurement error

**Fig. 4:**
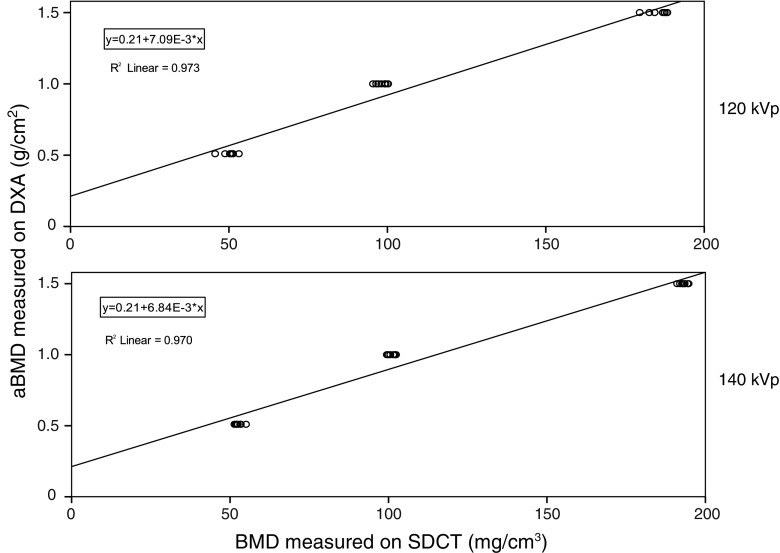
Comparison of BMD measurements on SDCT and aBMD measurements on DXA. Scatter plots with linear fit show a strong correlation. *aBMD* areal bone mineral density, *BMD* bone mineral density, *DXA* dual energy X-ray absorptiometry, *kVp* kilovoltage peak, *SDCT* dual-layer spectral detector computed tomography

At both 120 and 140 kVp, strong linear correlations (R^2^ = 0.973 and 0.970, respectively, P < 0.001) were found between BMD measurements on SDCT and aBMD measurements on DXA (Fig. 4). A positive intercept on the DXA-axis of 0.21 g/cm^2^ was found at both 120 and 140 kVp. The coefficients [95% CI] yielded β = 0.007 [0.007–0.008] at 120 kVp and β = 0.007 [0.006–0.007] at 140 kVp.

## Discussion

In this systematic phantom study, we showed that BMD can be quantified with an overall high accuracy on various dual-layer spectral detector CT protocols. In addition, a strong linear correlation was demonstrated between BMD measurements on SDCT and DXA. This indicates that SDCT could potentially open up the possibilities of BMD quantification on routinely acquired SDCT scans for any indication without exposing patients to additional ionizing radiation.

The American College of Radiology defined a BMD of <80 mg/cm^3^ as osteoporosis and 80–120 mg/cm^3^ as osteopenic [29]. In the (patho)physiological range of 50–200 mg/cm^3^ HA, we found an overall mean measurement error of 6% or less. This range of HA is a representative coverage of the amount of trabecular BMD clinically encountered at all age groups [24], and therefore important for testing our quantitative method before using it in clinical practice. In addition, scans were made using various settings to simulate clinically used abdominal, chest and spine SDCT imaging protocols. Overall small differences (≤5%) were observed between scan protocols, implying the possibility for follow-up of BMD, measured on SDCT scans made for different indications. An exception to this small difference of ≤5% is the 120 kVp 50 mAs protocol, the largest measurement error (-5.8 ± 3.4%) and largest mean difference between protocols (10%) was found for measurements made at this lowest dose protocol (Fig. 3). Measurements at this lowest dose protocol were significantly lower than all 140 kVp acquisitions protocols. Because no significant differences were found between BMD measurements at different iterative reconstruction levels, it is likely that results were only slightly influenced by noise. Increased measurement errors at this lowest dose protocol can be potentially explained by spectral separation. When scanning with a lower tube voltage, less high-energy photons are emitted and therefore the spectral overlap between the high- and low-energy spectra increases and spectral separation thus decreases. In addition, lowering the tube current decreases the number of photons emitted, thereby decreasing the number of photons that hit the detector. Both factors could influence the accuracy of the mass attenuation coefficient across monochromatic energies and thereby the accuracy of BMD quantification.

The influence of this decreased number of photons that hit the detector can be seen when comparing scan protocols, where an increase in mean measurement error is seen when lowering tube current (Table 2, Fig. 3). An exception is found for the 120 kVp 100 mAs protocol at 100 mg/cm^3^ and the 140 kVp 50 mAs protocol at 200 mg/cm^3^. However, a larger SD was also found for these protocols. Indicating that, despite a slightly less underestimated mean measurement error, accuracy was not evidently better. It remains unclear which factors caused these increased mean measurement errors. As described before, no significant differences were found when applying iterative reconstruction levels, indicating that it is likely that noise was of little influence. Interscan and intraobserver variation could be an influence, therefore these factors should be evaluated in future research.

Since the commercial introduction of DECT a decade ago, several studies have described the feasibility of BMD quantification [20–23, 30]. Wesarg and colleagues described that a 3D display of BMD distribution obtained with a dual source DECT gave more detailed information of focal bone solidity compared to BMD measured on DXA [20, 21]. However, measurement errors were not assessed. Wait et al. [23] described a root-mean-square error accuracy of at best of 9% when assessing BMD, expressed as K_2_HPO_4_ ranging from 0 to 600 mg/ml, in a phantom scanned with rapid kVp switching DECT. In accordance with our study, mass attenuation coefficients were obtained from the National Institute of Standards and Technology database to determine material density. In this way, no calibration phantom is needed, making (retrospective) BMD measurements on scans made for another indication than BMD possible. In a recent study Hofmann and colleagues presented a three-material decomposition method for BMD quantification on DECT, which they tested on the ESP [22]. They found a mean measurement error of about 3.5% over all HA concentrations and beam voltages, which is in accordance with the mean measurement error of -2.2 ± 3.3% we found over all HA concentrations and beam voltages in the present study. In addition, Hofmann et al. found an overestimation in HU scores obtained by quantitative CT when compared to their method and guidelines of the American College of Radiology, which underscores that DECT has better accuracy compared to quantitative CT [22].

In our study we observed a linear correlation between BMD measurements performed on SDCT and DXA. In accordance with Wait et al. [23], the linear correlation had a higher intercept at the DXA-axis (Fig. 4). A possible explanation for this higher intercept, and thus overestimation of DXA compared to SDCT, can be found in the fact that DXA also takes into account cortical bone, whereas our proposed SDCT method only takes into account trabecular bone. Surrounding structures and spinal changes that influence the DXA are ignored by the proposed SDCT method; while not evaluated in the current phantom setup, this may be of influence in a patient study. This effect of surrounding structures has been emphasized, leading to a decrease of correlation, by prior phantom, cadaver and patient studies [20, 21, 23].

In the present study we propose a relatively easy material decomposition method for BMD quantification using mass attenuation coefficient across monochromatic energies obtained from SDCT. The monochromatic image formation was performed on the raw data in the projection space, thereby effectively eliminating beam hardening artefacts [31]. Dual energy CT, using high monochromatic images (>95 keV), has been shown to successfully reduce metal artefacts in patients, while at low monochromatic energies metal artefacts were more enhanced [26, 27]. For our method we obtained monochromatic images at energy levels between 90 and 200 keV, suffering the least from metal artefacts. Together with the elimination of beam hardening and slice selective measurements, our proposed method may allow for BMD assessment of tissue surrounded by metal implants and thereby evaluate degeneration and assess the risk for (postoperative) fractures; however, further research on this topic is needed.

Our study has some limitations. We used a phantom setup and have not yet validated our method on patients. However, by using a phantom we were able to systematically test our quantification method in an optimally controlled setting with a clinically relevant range of known BMD concentrations. In addition, we were able to evaluate multiple scans protocols on an unmoved phantom, thereby minimizing interscan variation, which in a patient setting would add up to a high dose. Nonetheless, our method has to be verified in a patient study in future research. A second limitation is that we used a phantom without contrast medium, therefore the influence of contrast medium was not evaluated. Future research will have to address a three or multi-material decomposition, taking into account the attenuation coefficient of contrast medium. A third limitation is that we used a phantom that resembles the lumbar region, this could potentially introduce errors for the SDCT imaging protocols that simulate chest protocols. A fourth limitation is that we used a relatively small phantom, as the ESP represents a small adult person. One would expect more noise when larger phantoms (or patients) are imaged, which could potentially influence the accuracy in a negative way, especially for the lower dose protocols. However, one could argue that the noise encountered at 50 mAs in our phantom would be the same amount of noise encountered at a 100- or 200-mAs protocol in a larger patient. A fifth limitation is that we only evaluated the SDCT, therefore our results may be limited to the DECT technique described in this study.

In conclusion, we demonstrated that BMD can be accurately quantified with a novel dual-layer SDCT system using various acquisition protocols. Care should be taken when lowering the dose, as we found increased measurement errors and significant differences between other protocols for measurements made at the lowest dose protocol. A strong linear correlation between all SDCT measurements and DXA measurements was obtained. Our results potentially opens up the possibilities for osteoporosis evaluation and opportunistic screening on SDCT scans made for other clinical indications. However, patient studies are needed to extend and translate our findings.

## Abbreviations

aBMD: Areal bone mineral density
ANOVA: Analysis of variance
BDC: Bone density calibration phantom
BMD: Bone mineral density
CT: Computed tomography
CTDIvol: Volumetric CT dose index
DECT: Dual energy computed tomography
DXA: Dual energy X-ray absorptiometry
ESP: European spine phantom
HA: Calcium hydroxyapatite
keV: Kilo electron voltage
kVp: Kilovoltage peak
mAs: Milliampere second
NIST: National institute of standards and technology
ROI: Region of interest
SBI: Spectral-based images
SDCT: Dual-layer spectral detector computed tomography
QCT: Quantitative computed tomography
